# SLC26A9 promotes colorectal tumorigenesis by modulating Wnt/β-catenin signaling

**DOI:** 10.1038/s41420-024-01888-6

**Published:** 2024-03-09

**Authors:** Minglin Zhang, Zhiyuan Ma, Zhiqiang Yi, Hu Wang, Jiaxing Zhu, Guorong Wen, Hai Jin, Jiaxing An, Zilin Deng, Biguang Tuo, Taolang Li, Xuemei Liu

**Affiliations:** 1 Department of Gastroenterology, Digestive Disease Hospital, Affiliated Hospital of Zunyi Medical University, Zunyi, China; 2 Department of General Surgery, Affiliated Hospital of Zunyi Medical University, Zunyi, China

**Keywords:** Tumour biomarkers, Prognostic markers

## Abstract

Solute carrier family 26 member 9 (SLC26A9) is a member of the Slc26a family of multifunctional anion transporters that functions as a Cl^-^ channel in parietal cells during acid secretion. We explored the role of SLC26A9 in colorectal cancer (CRC) and its related mechanisms through clinical samples from CRC patients, CRC cell lines and mouse models. We observed that SLC26A9 was expressed at low levels in the cytoplasm of adjacent tissues, polyps and adenomas but was significantly increased in colorectal adenocarcinoma. Moreover, increased levels of SLC26A9 were associated with a high risk of disease and poor prognosis. In addition, downregulation of SLC26A9 in CRC cells induced cell cycle arrest and apoptosis but inhibited cell proliferation and xenograft tumor growth both in vitro and in vivo. Mechanistic analysis revealed that SLC26A9 was colocalized with β-catenin in the nucleus of CRC cells. The translocation of these two proteins from the cytoplasm to the nucleus reflected the activation of Wnt/β-catenin signaling, and promoted the transcription of downstream target proteins, including CyclinD1, c-Myc and Snail, but inhibited the expression of cytochrome C (Cyt-c), cleaved Caspase9, cleaved Caspase3 and apoptosis-inducing factor (AIF). CRC is accompanied by alteration of epithelial mesenchymal transition (EMT) markers. Meanwhile, further studies showed that in SW48 cells, overexpressing SLC26A9 was cocultured with the β-catenin inhibitor XAV-939, β-catenin was downregulated, and EMT was reversed. Our study demonstrated SLC26A9 may be responsible for alterations in the proliferative ability and aggressive potential of CRC by regulating the Wnt/β-catenin signaling pathway.

## Background

Colorectal cancer (CRC) is a common malignant tumor and a major health threat worldwide [1, 2]. The specific pathogenesis of CRC needs to be further explored. In normal colorectal mucosal tissue, ion channels and transporters play an important role in maintaining hydroelectrolyte and acid-base homeostasis. However, dysfunction of these ion channels and transporters leads to the occurrence of CRC [3]. Therefore, we further explored the molecular mechanism of CRC and provided a scientific theoretical basis for the pathogenesis and therapeutic significance of CRC.

SLC26A9, a member of the SLC26A family, is essentially a multifunctional anion transporter that functions as a Cl^-^ channel [4], Cl^−^/HCO_3_^−^ exchanger [5–7], or sodium transporter [8] and is highly expressed in the lungs [9], stomach [4] and kidneys [10] but with low expression levels in the colorectal epithelium [4, 11]. Our previous study found that downregulation of SLC26A9 is involved in the occurrence of gastric cancer, which is the first evident to show that SLC26A9 is involved in the occurrence of gastric cancer [12]. Therefore, the mechanism of SLC26A9 in carcinogenesis has never been explored and needs further study.

Therefore, we detected the expression of SLC26A9 in clinical CRC samples by Immunohistochemistry (IHC) and western blot (WB) and revealed the effects of SLC26A9 on the biological behavior of CRC cells in vitro and tumor growth in vivo. We also present evidence for the interaction of SLC26A9 with β-catenin.

## Results

### SLC26A9 is upregulated in CRC and associated with poor prognosis

**Fig. 1:**
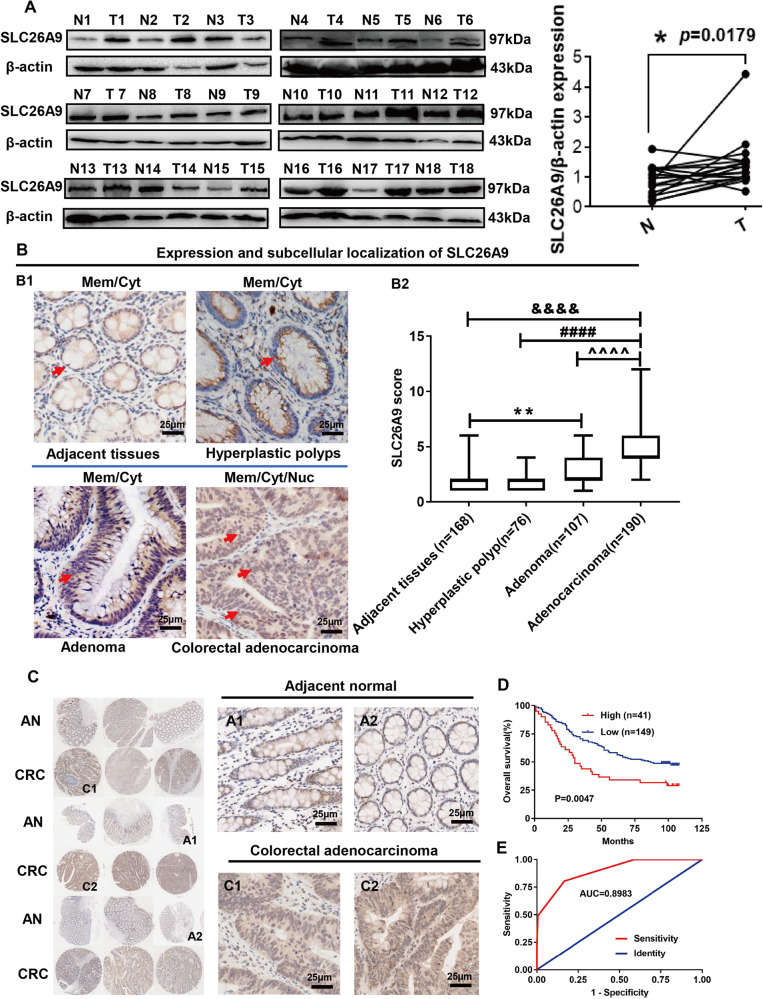
Upregulation of SLC26A9 is associated with the development and progression of human CRC as well as a poor prognosis. **A** SLC26A9 protein expression in adjacent tissues. and CRC tissues was detected by Western blots, *p < 0.05, compared with adjacent tissues, *n* = 18 in each series. **B** IHC analysis showed that SLC26A9 is progressively upregulated from adjacent tissues to polyps, adenomas and CRC tissues, with strong expression in the nucleus, cytoplasm and cell membrane of CRC tissues. The left panel shows representative images, and the right panel shows a comparison of SLC26A9 expression levels between groups. **p < 0.01 and ^&&&&^*p* < 0.0001, compared with adjacent tissues; ^^^^*p* < 0.0001, compared adenocarcinoma with adenomas groups; ^####^*p* < 0.0001, compared adenocarcinoma with hy*p*erplastic polyp groups, scale bars = 25 μm. **C**, **D** Upregulation of SLC26A9 in CRC determined by IHC in a tissue microarray is related to a poor prognosis. The left panel shows representative images of SLC26A9 expression in CRC and adjacent tissues. The right panel shows the patient prognosis comparison between groups, scale bars = 25 μm. **E** ROC analysis of the prognostic sensitivity and specificity for CRC patients and detected by the expression of SLC26A9 in CRC tissues. N adjacent normal, T Tumor, AN adjacent normal, CRC colorectal cancer, Mem Membrane, Cyt Cytoplasm, Nuc nucleus.

Western blotting analysis showed that compared with adjacent tissues, SLC26A9 was highly upregulated in CRC (Fig. 1A). Moreover, IHC analysis showed that SLC26A9 was localized in the cytoplasm and cell membrane of adjacent tissues with low expression levels (*n* = 168), but increased in polyps (*n* = 76) and adenomas (*n* = 107) and was especially significantly upregulated in colorectal adenocarcinoma (*n* = 190), with translocation to the nucleus (Fig. 1B). This study showed that the expression of SlC26A9 gradually increased during the process of CRC formation (polyps, adenomas and CRC).

Furthermore, we analyzed the tissue chips of 190 CRC tissues and 168 adjacent tissues by IHC, analyzed the relationship between SLC26A9 expression and patient survival, calculated the protein expression of SLC26A9 in the tumor (Fig. 1B), and classified the patient population according to the total immunohistochemistry score: SLC26A9 high and SLC26A9 low. The patients in the SLC26A9 high-expression group had lower overall survival than those in the SLC26A9 low-expression group (Fig. 1C, D). In addition, the ROC curve indicates that SLC26A9 can be used to diagnose and monitor the clinical treatment effect and has potential clinical significance as a tumor marker (Fig. 1E).

Finally, we analyzed SLC26A9 expression in 190 CRC tissue microarray samples. SLC26A9 overexpression was associated with T stage (*p* < 0.05), lymph node metastasis (*p* < 0.05), distant metastasis (*p* < 0.01), TNM stage (*p* < 0.05), and Duke stage of CRC but was not correlated with age, sex or tumor size (Supplementary Figure 1A–H). These data suggested that SLC26A9 can be used as a potential prognostic biomarker in CRC patients.

### SLC26A9 promotes CRC cell proliferation and migration in vitro

**Fig. 2:**
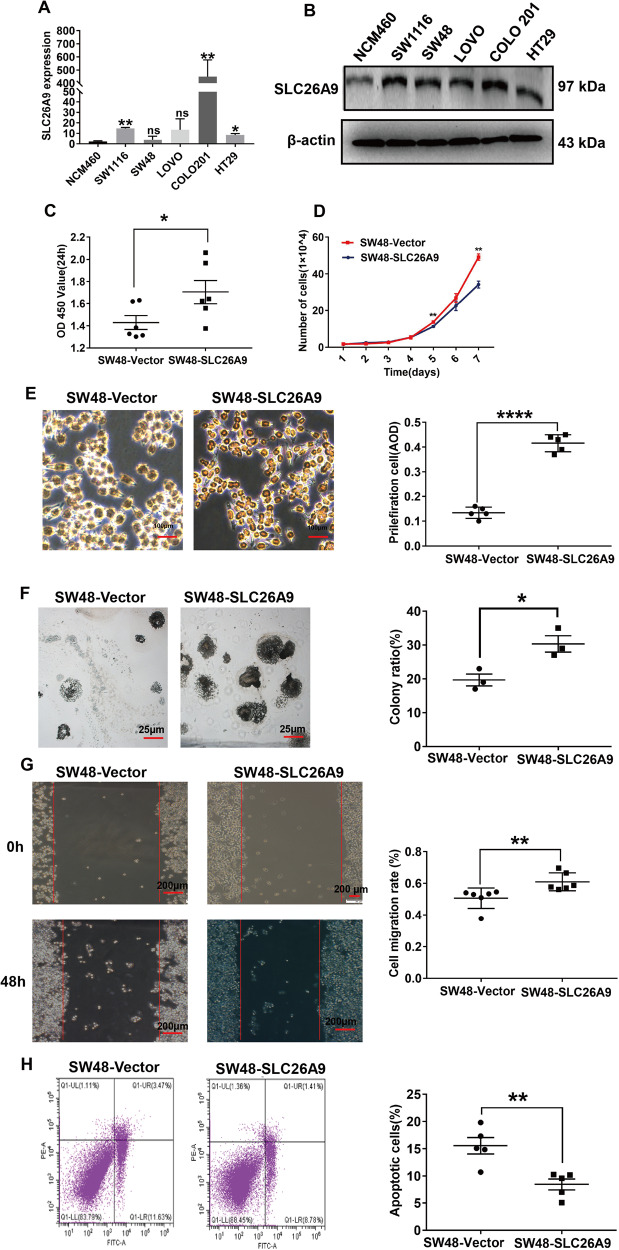
Effect of SLC26A9 overexpression in SW48 cells on their biological behavior in vitro. SLC26A9 mRNA and protein were detected in CRC cell lines by **A** RT‒qPCR and **B** western blotting analysis. Each bar represents the mean ± SD. **C** Cell proliferation detected by CCK-8 assay, **D** cell growth determined by the growth curve assay, **E** Ki-67 staining, scale bars = 100 μm, **F** colony formation experiment, scale bars = 25 μm, **G** Cell migration was detected by wound healing assay, scale bars = 200 μm, **H** cell apoptosis determined by flow cytometry. *p < 0.05, **p < 0.01 and ****p < 0.0001, ns not significantly different, compared with relevant controls, *n* = 3–6 in each series.

To research the biological function of SLC26A9 in CRC, we detected the expression of SLC26A9 in various CRC cell lines (SW1116, SW48, LOVO, COLO 201 and HT29) by RT-qPCR and WB. Consistent with human data, SLC26A9 was expressed at low levels in the colon mucosal cell line NCM460 but upregulated in all CRC cell lines at both the mRNA and protein levels, with the lowest expression observed in SW48 cells and the highest expression observed in COLO 201 cells (Fig. 2A, B). We selected SW48 and COLO 201 cells for further analysis of biological behavior and molecular mechanisms. We established stable overexpression in SLC26A9 cells (SW48-SLC26A9) and stable SLC26A9 knockdown in COLO 201 cells (COLO 201-shSLC26A9) to ensure the role of SLC26A9 in proliferation and migration.

RT-qPCR and Western blot analysis showed that SLC26A9 expression was upregulated in SW48-SLC26A9 cells (Supplementary Figure 2A, B). A CCK-8 assay was used to detect the viability of SW48 cells, and the results showed that overexpression of SLC26A9 promoted the viability of SW48 cells (Fig. 2C). Furthermore, overexpression of SLC26A9 increased the proliferation ability of CRC cells, which was detected by growth curve experiments and Ki-67 staining assay (Fig. 2D, E), as well as increased the viability and clonogenic ability and promoted the migration ability of CRC cells (Fig. 2F, G). However, SLC26A9 overexpression reduced the total number of apoptotic SW48 cells, as determined by flow cytometry analysis (Fig. 2H).

### SLC26A9 modulates cell apoptosis and EMT-induced cancer stem cell (CSC) phenotypes

**Fig. 3:**
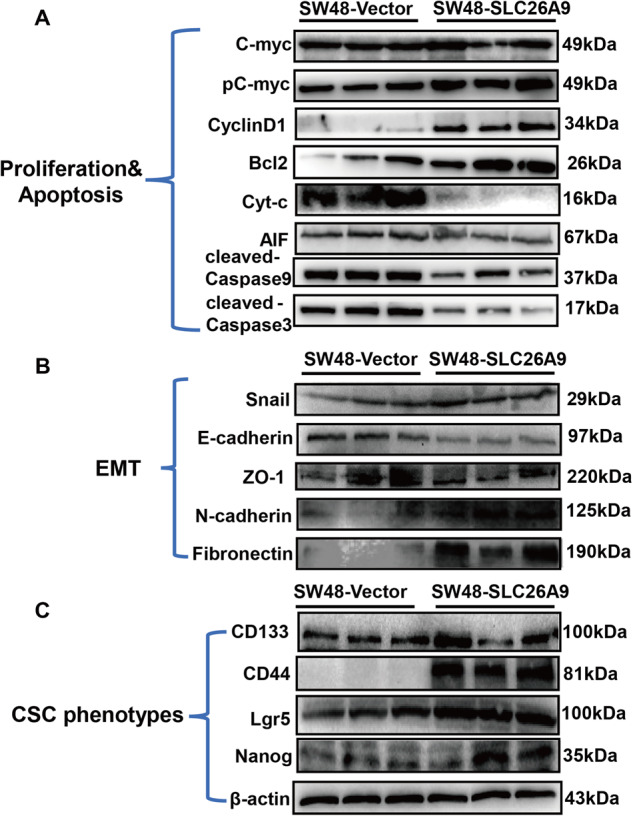
SLC26A9 modulates cell apoptosis and EMT-induced CSC phenotypes. Multiple markers of cell proliferation and apoptosis (**A**), EMT (**B**) and CSC phenotypes (**C**) were detected in SW48 cells with and without SLC26A9 overexpression by Western blot experiments.

The disruption of the balance between apoptosis and proliferation of colorectal epithelial cells leads to the occurrence of CRC, and the formation of CRC is related to the regulation of the Wnt signaling pathway [13]. Our data showed that overexpression of SLC26A9 in SW48 cells increased expression of pc-Myc, and upregulation of CyclinD1 and Bcl2, whereas Cyt-c, cleaved Caspase 9, cleaved Caspase 3 and AIF were downregulated, indicative of the suppression of caspase-dependent and caspase-independent apoptosis (Fig. 3A). Moreover, at the protein level, it was found that the upregulation of SLC26A9 led to the downregulation of E-cadherin and ZO-1 expression, but the expression of Snail, N-cadherin and Fibronectin was upregulated, which is related to the EMT phenotype (Fig. 3B). During the EMT process, epithelial cells dedifferentiate to mesenchymal cells, and the EMT-induced CSC phenotype contributes to the development of CRC [14]. We detected the expression of surface markers, including CD44, CD133, Lgr5 and Nanog, when compared with the vector group (Fig. 3C). These results demonstrated that upregulation of SLC26A9 alters cell phenotypes.

### Inhibiting SLC26A9 expression in CRC cells inhibits neoplastic growth both in vitro and in vivo

**Fig. 4:**
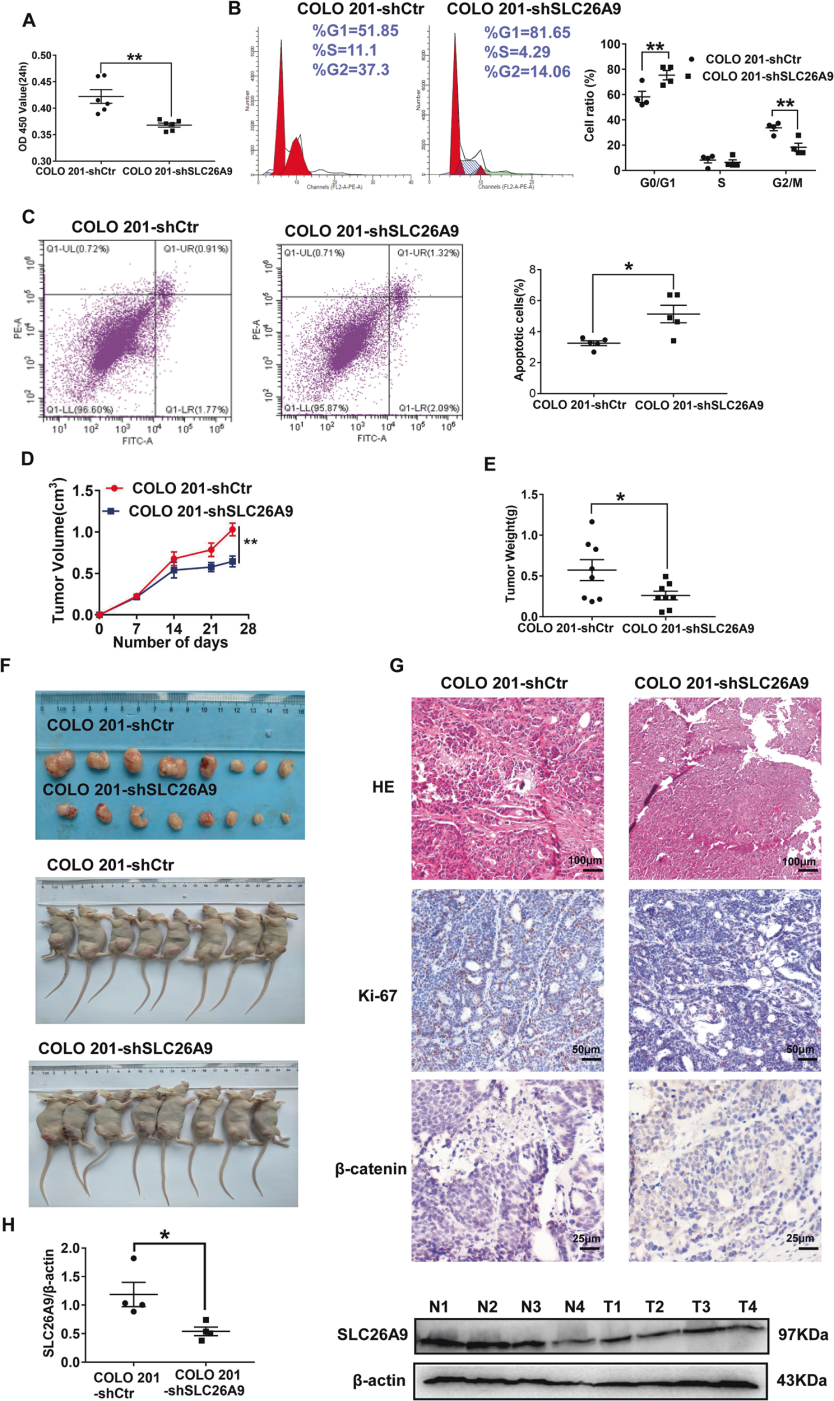
Inhibiting SLC26A9 expression in colon cancer cells inhibits neoplastic growth both in vitro and in vivo. Cell proliferation was assessed by CCK-8 assay (**A**); cell cycle (**B**). **C** Cell apoptosis was determined by flow cytometry analysis. **D**–**F** Flank tumor xenograft tumor development after subcutaneous injection (*n* = 8 mice per group) was monitored for COLO 201 cells, Tumor volume and tumor weight of COLO 201-shSLC26A9 cells 25 days after inoculation in nude mice. **G** HE staining of paraffin sections of tumors after injection of COLO 201 cells transfected with shSL26A9 at 25 days, scale bars = 100 μm. Tumors were evaluated for β-catenin and Ki-67 expression by immunohistochemistry, scale bars = 50 or 25 μm. **H** WB for SLC26A9 in tumors from 4 mice from each group (T1, T2, T3 and T4), normalized to β-actin as a control. For graphs, data represent the mean ± SD, *p < 0.05; **p < 0.01, compared with relevant controls, *n* = 4-6 in each series.

To evaluate the effect of SLC26A9 reduction in CRC cells, we constructed silent stable strains in CRC cells by silencing the expression of SLC26A9 by lentivirus. RT-qPCR and western blot analysis proved a significant decrease in SLC26A9 expression in COLO 201-shSLC26A9 cells compared with control cells (COLO 201-shCtr) (Supplementary Fig. 2C, D). Compared with the COLO 201-shCtr groups, SLC26A9 silencing significantly suppressed cell proliferation ability, inhibited G1 phase into S phase and G2/M phase, and suppressed transcription (Fig. 4A, B), followed by increased total apoptotic cells in COLO 201 by flow cytometry analysis (Fig. 4C). However, due to the semi adherent ability of COLO 201, cell migratory and invasive ability analyses were not performed.

Next, we surveyed the impact of SLC26A9 on neoplasm growth in vivo. For xenograft growth assays, we injected COLO 201-shSLC26A9 cells and COLO 201-shCtr cells subcutaneously into the armpit of nude mice (*n* = 8 in each group). Subcutaneous tumor growth was palpable on day 7 after injection. At day 25, tumor size was significantly smaller in the COLO 201-shSLC26A9 group. The subcutaneous tumor weight was lower in the COLO 201-SLC26A9 group than in the control group (Fig. 4D–F). Hematoxylin-eosin (H&E) staining revealed that the histological features of the mass were similar to those of human CRC (Fig. 4G). The results showed that SLC26A9 silencing had an effect on cell proliferation. In addition, we found that the expression of SLC26A9, Ki-67 and β-catenin was downregulated in the COLO 201-shSLC26A9 group (Fig. 4G, H). Moreover, SLC26A9 silencing in COLO 201 cells decreased cell proliferation and reversed EMT-induced CSC phenotypes (Supplementary Fig. 3B–D).

### SLC26A9 regulates the Wnt/β-catenin signaling pathway

**Fig. 5:**
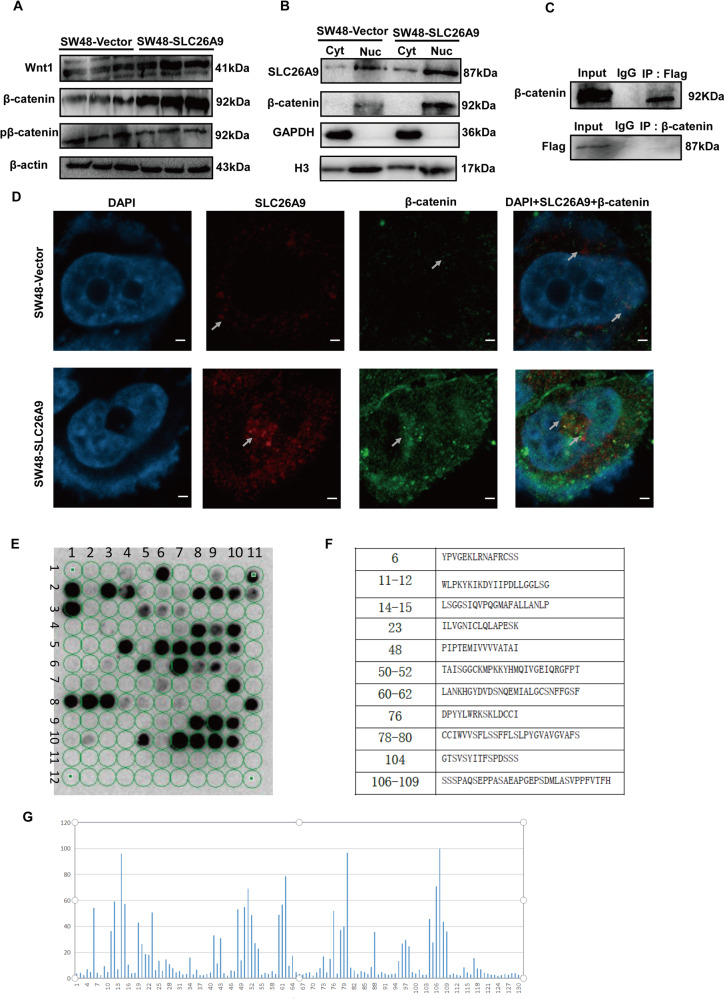
SLC26A9 positively regulates the Wnt/β-catenin signaling pathway in CRC cells. **A** Western blotting showing the expression levels of Wnt/β-catenin signaling pathway-related proteins Wnt1, β-catenin and pβ-catenin. **B** The expression of SLC26A9 and β-catenin was upregulated in the nucleus by cell nucleus and cytoplasm extraction. **C** Flag coimmunoprecipitates with β-catenin in CRC cells. immunoprecipitated with Flag antibody or control, IgG and detected with β-catenin antibody, immunoprecipitated with β-catenin antibody or control IgG and detected with SLC26A9 on western blot. **D** Immunofluorescence staining showing the presence and localization of SLC26A9 and β-catenin in the cytoplasm and nucleus. The white arrow shows the position of protein expression. Original magnification: 1260×. **E**, **F** Multiarray microarray showed that 11 different peptide fragments on the SLC26A9 protein-derived peptide array chip interacted with β-catenin and showed strong binding affinity. **G** Gray value analysis of the binding site between the SLC26A9-derived polypeptide array and the reactive protein β-catenin.

β-catenin is broken down from the cytoplasmic complex and activated via the WNT signaling pathway, migrating from the cytoplasm to the nucleus for subsequent transcriptional activation of several genes involved in proliferation, apoptosis and EMT-induced CSC phonotypes [15–19]. Then, we investigated the possible oncogenic pathways that may involve the function of SLC26A9. We found that overexpression of SLC26A9 in the SW48 cell line led to a sharp increase in the expression of Wnt 1 and β-catenin and decrease in the expression of pβ-catenin (Fig. 5A). SLC26A9 and β-catenin were determined by separating cytoplasmic nucleocapsid protein, and subcellular components were extracted from the SW48 cell line to analyze whether SLC26A9 and β-catenin were present. We found that SLC26A9 was predominantly expressed in the nucleus and a small amount in the cytoplasm, and β-catenin was also increased in the nucleus in the SW48 cell line. Furthermore, overexpression of SLC26A9 resulted in translocation of SLC26A9 and β-catenin from the cytoplasm to the nucleus in the SW48 cell line (Fig. 5B). Additionally, the results of coimmunoprecipitation showed that SLC26A9 and β-catenin interact with each other (Fig. 5C). Importantly, immunofluorescence colocalization showed that the re-expression of SLC26A9 in SW48 cells resulted in the colocalization and translocation of SLC26A9 and β-catenin into the nucleus compared with the control group (Fig. 5D). Additionally, SLC26A9 silencing in COLO 201 inhibited the expression of the Wnt/β-catenin signaling pathway, resulting in inhibition of the expression of proliferation- and EMT-induced CSC phenotype target proteins (Supplementary Figure 3A–D). Taken together, these data showed the positive interaction between SLC26A9 and β-catenin. To further illustrate our speculation, peptide microarray analysis was performed according to the sequence of the SLC26A9 protein. The data showed that 11 different peptide fragments on the SLC26A9 protein-derived peptide array chip interacted with β-catenin and showed strong binding affinity, including 6, 11–12, 14–15, 23, 48, 50–52, 60–62, 76, 78–80, 104, and 106–109 (Fig. 5E–G). These results supported that SLC26A9 positively interacted with β-catenin and translocated from the cytoplasm to the nucleus to induce the development of CRC.

### XAV-939/CHIR-99021 regulates the response of the Wnt/β-catenin signaling pathway

**Fig. 6:**
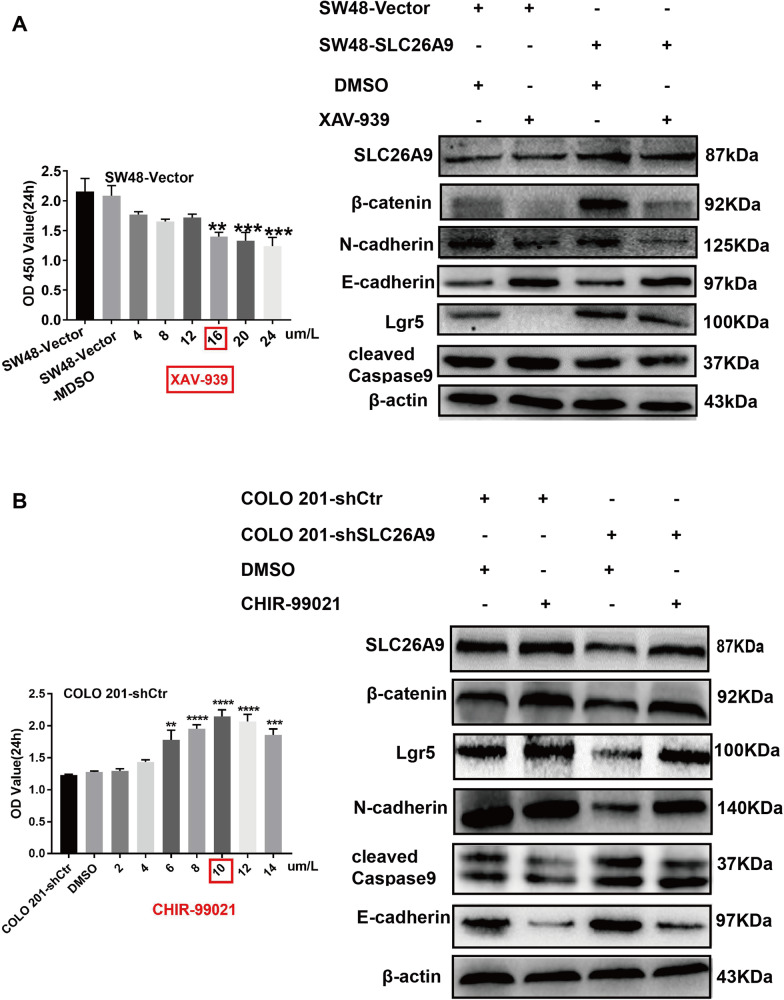
SLC26A9 mediates EMT-induced CSC by activating the Wnt/β-catenin signaling pathway. **A**, **B** The optimal inhibitory concentrations of the β-catenin inhibitor XAV-939 and the specific agonist CHIR-99021 are shown in the left panel. SW48 cells with and without SLC26A9 overexpression were treated with XAV-939 (16 μm/L) for 24 h (**A**). COLO 201 cells with and without SLC26A9 inhibition were treated with CHIR-99021 (10 μm/L) for 24 h (**B**), and the indicated proteins of the EMT and CSC phenotypes were then assayed by western blotting. Bar graph on the left, **A**, **B**, the data are presented as the mean ± SEM. **p < 0.01, ***p < 0.001, ****p < 0.0001 compared with relevant controls, *n* = 3 in each series.

We further investigated whether SLC26A9 promotes cell proliferation and EMT-induced CSC phenotypes through the Wnt/β-catenin signaling pathway in CRC. The β-catenin-specific inhibitor XAV-939 at 16 μm/L with the highest inhibitory effect decreased the expression of β-catenin, SLC26A9, Lgr5 and N-cadherin but increased the expression of cleaved Caspase 9 and E-cadherin in SW48-SLC26A9 cells (Fig. 6A), indicating that XAV-939 disrupted SLC26A9 expression and the Wnt/β-catenin signaling pathway, resulted in the promotion of apoptosis, and reversed EMT-induced CSC phenotypes. In contrast, the β-catenin-specific agonist CHIR-99021 at 10 μm/L, with the highest agonist effect, increased the expression of β-catenin, SLC26A9, Lgr5 and N-cadherin and decreased the expression of cleaved Caspase 9 and E-cadherin in SLC26A9-silenced COLO 201 cells compared with the control group (Fig. 6B). Overall, these results suggest that SLC26A9 may interact with β-catenin to jointly activate the Wnt/β-catenin signaling pathway and initiate the transcription of downstream target genes, leading to the development and progression of CRC.

## Discussion

In this study, we revealed the carcinogenic effect of SLC26A9 in CRC. We demonstrated that (i) SLC26A9 expression was significantly upregulated in CRC and correlated with poor prognosis in clinical samples; (ii) SLC26A9 overexpression promotes proliferation and migration, leading to malignant transformation of CRC cells; and (iii) SLC26A9 may interact with β-catenin to participate in the activation of the Wnt/β-catenin signaling pathway and initiate the transcription of downstream target genes. The results illustrated that SLC26A9 may provide a new target for effective clearance of human CRC. SLC26A9, a member of the SLC26A family, is essentially a multifunctional anion transporter. In humans, SLC26A9 encodes 791 amino acids and is located on chromosome 1 [9]. SLC26A9 (human)/Slc26a9 (mouse) is expressed at high levels in the stomach [4, 20], but at low levels in the duodenum, pancreas and colorectal epithelium [4, 11, 21]. We found that SLC26A9 functions as Cl^-^ channel and plays an important role in gastric acid secretion and is also involved in the regulation of cell proliferation and migration, as well as the formation of gastric cancer [11, 12]. In normal colorectal mucosal tissue, ion channels and transporters play an important role in maintaining hydroelectrolyte and acid-base homeostasis. However, dysfunction of these ion channels and transporters leads to the occurrence of CRC [3]. Currently, intracellular Cl^-^ ions are considered to be an important signaling target [22–24], and high intracellular levels of Cl^-^ regulate cell proliferation and growth [22]. It has been reported that Cl^-^ channels upregulate Wnt/β-catenin pathway-related target genes in CRC cells [25]. Moreover, another epithelial Cl^-^ channel, CFTR is related to an increased incidence of colon cancer [26–28]. Previous studies showed that CFTR is positive correlation with SLC26A9 in the cystic fibrosis related diseases [11, 29, 30]. However, whether SLC26A9 is implicated in CRC tumorigenesis has never been explored.

In the present study, our data showed that SLC26A9 promoted the occurrence and development of CRC, and its expression was significantly increased in the protein analysis of clinical CRC samples, in CRC cells, and in The Cancer Genome Atlas (TCGA) database (Supplementary Fig. 4). Clinically, we confirmed that SLC26A9 was upregulated in human CRC tissues and associated with poor prognosis (Fig. 1). These data suggest that SLC26A9 is a prognostic biomarker for aggressiveness in CRC patients. SLC26A9 overexpression in CRC cells increased their proliferative and migratory properties in vitro (Fig. 2). In contrast, SLC26A9 silencing promoted the apoptosis of human CRC cells but inhibited their migratory functions in vitro and reduced tumor growth in vivo (Fig. 4), suggesting that SLC26A9 may have a proliferative and migratory promoting effect during CRC progression. β-catenin is a key regulatory molecule of the typical Wnt signaling pathway and plays an important role in the regulation of a variety of cellular processes, including cell proliferation, survival, migration, invasion, polarity, differentiation and stem cell self-renewal in colorectal carcinogenesis [16, 31].

Previously, our data showed that gene microarray assay demonstrated that SLC26A9 gene modification resulted in many singling pathways alterations, including Wnt/β-catenin signaling, P53 singling pathway, SHH signaling pathway, MAPK signaling pathway and so on [12], Furthermore, the Wnt/β-catenin pathway is activated and β-catenin degradation is reduced, resulting in nuclear accumulation of β-catenin as well as transcription of downstream target genes in the development of CRC as reported [32]. Additionally, β-catenin is an important EMT factor that can change the characteristics of epithelial cancer cells so that they can invade surrounding tissue and function as a key regulatory factor of the CSC phenotype, providing clues to the nature of tumor heterogeneity [33, 34]. However, whether Wnt/β-catenin signaling alteration in the development of CRC by upregulation of SLC26A9 is never investigated. Notably, we demonstrated that the SLC26A9 protein colocalized with β-catenin and translocated from the cytoplasm to the nucleus through protein–protein interactions, reflecting the activation of Wnt/β-catenin signaling (Fig. 5, and Supplementary Fig. 3A). Moreover, peptide microarray analysis was performed according to the sequence of the SLC26A9 protein. The data showed that 11 different peptide fragments on the SLC26A9 protein-derived peptide array chip interacted with β-catenin and showed strong binding affinity, including 6, 11–12, 14–15, 23, 48, 50–52, 60–62, 76, 78–80, 104, and 106–109 (Fig. 5E–G). These results demonstrated that SLC26A9 positively interacted with β-catenin. However, the precise mechanism needs to be further investigated.

We speculated that SLC26A9 enhanced canonical Wnt signaling in CRC cells based on the following events: (i) it increased the expression of pc-Myc, Bcl2, CyclinD1 and Snail but decreased that of AIF, cleaved Caspase 9 and cleaved Caspase 3, ultimately resulting in suppression of caspase-dependent and caspase-independent apoptosis; (ii) Snail expression was upregulated to promote EMT transformation, and E-cadherin expression was downregulated to promote migration and invasion; and (iii) EMT, as a key regulatory factor of the CSC phenotype, provided clues to the nature of tumor heterogeneity. Upregulation of SLC26A9 resulted in EMT-induced CSC proteins, including CD44, CD133, Lgr5 and Nanog enrichment (Fig. 3C). These results demonstrated that upregulation of SLC26A9 alters cell phenotypes and contributes to colorectal carcinogenesis. To further illustrate that the Wnt/β-catenin signaling pathway regulates SLC26A9-mediated EMT, we used the β-catenin inhibitor XAV-939 to detect the role of SLC26A9. Our results revealed that the β-catenin inhibitor XAV-939 decreased the expression of β-catenin, SLC26A9, Lgr5 and N-cadherin but increased the expression of cleaved Caspase 9 and E-cadherin in SW48-SLC26A9 cells (Fig. 6A), indicating that XAV-939 disrupted SLC26A9 expression and the Wnt/β-catenin signaling pathway, resulted in the promotion of apoptosis, and reversed EMT-induced CSC phenotypes. Overall, these results indicate that SLC26A9-induced EMT is dependent on the Wnt/β-catenin signaling pathway in CRC. However, whether the expression of SLC26A9 alone is sufficient to achieve Wnt target gene activation or whether its phosphorylation or other activities are also involved remains to be determined in the future with effective and specific inhibitor.

## Conclusions

This study can help to understand the underlying molecular mechanism by which SLC26A9 upregulation in human CRC promotes neoplasm initiation and progression by activating the Wnt/β-catenin signaling pathway. These findings may provide a theoretical basis for CRC diagnosis and therapeutic targets in the future. To understand in detail the specific mechanism of SLC26A9 in cancer induction, it is necessary to elucidate the genetic basis of abnormal SLC26A9 expression in CRC through further molecular mechanism studies.

## Methods

### Tissue microarray and human samples

The expression level of SLC26A9 in CRC tissues was detected by the tissue chip technique (TMA). A tissue microarray containing 190 cases of multiple human CRC tissues was purchased from Shanghai Outdo Biotech (catalog nos. HColA180Su15 and HRec-Ade180Sur-03, Shanghai, China). Demographic and clinicopathological data, including clinical stage (according to the American Joint Committee on Cancer staging system) and survival data, were provided by the manufacturer. Human samples, including colorectal hyperplastic polyps (*n* = 76) and adenomas (*n* = 107), were collected from the Department of Pathology, Affiliated Hospital of Zunyi Medical University (Zunyi, China) from May 2018 to May 2021. The study was carried out in accordance with the Second Declaration of Helsinki and was approved by the Human Experimentation Committee of the Affiliated Hospital of Zunyi Medical University. All biopsy patients signed written informed consent. Immunohistochemical images were captured with a BX60 microscope (Olympus, Tokyo, Japan). The staining strength score was as follows: 0 (no staining), 1 (weak staining), 2 (moderate staining), and 3 (strong staining). and the extent of stained cells (0% = 0, 1–24% = 1, 25–49% = 2, 50–74% = 3, 75–100% = 4). Final immunohistochemical score = immune response strength score multiplied by stained cell score, 0 (lowest score) to 12 (highest score). An immunohistochemical score ≥6 was considered high expression, and an immunohistochemical score < 6 was considered low expression.

### Immunohistochemistry

EDTA was used for antigen repair and goat serum was used for antigen blocking. Primary antibodies against SLC26A9 (1:50 dilution ratio) and β-catenin (1:200 dilution ratio) were incubated with the slides at 4 °C overnight. Following this, the slides were incubated with a secondary antibody at 37 °C for 30 min. DAB (3,3′-diaminobenzidine) was used for visualization, and hematoxylin was used for counterstaining. Microscopic observation record.

### Ki-67 staining assay

Six-well plates to prepare cell slides, washing with PBS, fixed with paraformaldehyde, permeabilization with 0.1–0.25% Triton X-100, Ki-67 (1:50 dilution ratio) was incubated with the slides at 4 °C overnight, washed three times with PBS. add secondary antibody and incubate for 1 h at room temperature, DAB color development, the microscope takes pictures, statistical analysis.

### Western analysis

Protein separation, protein quantification and Western blot analysis have been described in previous study [12, 35], and the list of primary antibodies is shown in Supplementary Table 1. Cytoplasmic and Nuclear Proteins Extraction Kit (TransGen Biotech) as previously described [36].

### RNA extraction and RT‒qPCR

The methods of total mRNA extraction, mRNA reverse transcription into cDNA, and cDNA amplification were described in previous study [12, 37]. The sequences of different genes are shown in Supplementary Table 2.

### Cell culture

CRC cell lines (LOVO and SW1116) were purchased from the Cell Bank of the Type Culture Collection of the Chinese Academy of Sciences, Shanghai Institute of Cell Biology, Chinese Academy of Sciences (Shanghai, China). Normal colonic mucosal NCM460 cells and CRC cell lines (SW48 and COLO 201) were purchased from the Cell Bank of Type Culture Collection of BeNa Culture Collection (from the American Type Culture Collection (ATCC, Manassas, VA, USA). The cells were cultured in DMEM with 10% FBS and then placed in 37 °C humidified incubators with 5% CO_2._

### Construction of lentiviral transfection of SW48 and COLO201 cells

SW48 and COLO201 cells were transfected with lentivirus carrying the SLC26A9 gene fragment, shRNA, or empty vector (Hanbio Biotechnology, China). A stable strain was selected by puromycin. RT‒qPCR and western blot analyses were performed to validate the transfection efficiency.

### Cell counting Kit-8 (CCK-8) assay

Cell proliferation was analyzed using Cell Counting Kit 8 (CCK8, Beyotime Institute of Biotechnology, Shanghai, China). In the 96-well plate, the inoculated cell density was as follows: 24 h:3000 cells/well, 48 h:1500 cells/well, 72 h:1000 cells/well. Ten microliters of CCK8 solution were added to each well at 24, 48 and 72 h and incubated for 2 h. The absorbance (OD values) at 450 nm was measured to assess the number of viable cells.

### Colony formation assay

Colony formation assays were performed in 6-well culture plates. Plating cell density: 3 × 10^2^/well, cultured in CO_2_ incubator at 37 °C for 12 days, 6-well plates were removed, washed twice with PBS, fixed. Observation of the number of cell masses formed under a microscope (≥50 cells).

### Growth curve assay (cell counting method)

The transfected cells were collected and seeded into 7 24-well plates at a rate of 3 × 10^4^ cells/well. After 24 h, the number of cells was counted, and then one plate was randomly removed for counting every 24 h. The growth curve was drawn with the number of cells per unit (number of cells ×10^4^/ml) as the ordinate and time as the abscissa.

### Wound healing assay

Lentivirus-transfected SW48 cells were inoculated in a 24-well plate. When the cells were 80% confluent, a uniform scratch was made in the center of the hole with a 10 μl pipette head as a mark. Different transfected cells were photographed at 0, 24 and 48 h, and scratch healing was quantified.

### Cell apoptosis assays

Apoptosis assays were performed using SW48-vector and SW48-SLC26A9, COLO 201-shCtr and COLO 201-shSLC26A9 expression cells using the Annexin V-PE apoptosis detection kit (eBioscience, Vienna, Austria) according to the manufacturer’s instructions. The experiment was performed with five replicates for each cell line.

### Cell cycle analysis

The SLC26A9-shCOLO201 stable strain was constructed and inoculated into a 6-well plate. After 24 h, the culture was washed with PBS 3 times and fixed with 75% alcohol for 24 h. The experiment was conducted according to the instructions of The Cell Cycle and Apoptosis Analysis kit (Beyotime, Jiangsu, China). Subsequently, cell cycle analysis was performed using a flow cytometer (BD Biosciences, New Jersey, USA).

### Hematoxylin and eosin (H&E) staining

Tissue specimens were collected, fixed in 10% formalin for 2 h, dehydrated, embedded in paraffin, sliced, dewaxed with xylene, and treated with a graded ethanol series. Next, the sections were stained with H&E and observed using an IX53 microscope (Olympus).

### Chemicals

Cells in logarithmic growth phase were inoculated into 6-well plates. Routine culture. When the cells were 50-80% confluent, the serum-free medium was replaced, and the cells were starved for 24 h. XAV-939 was added at a working concentration of 16 μm/L. After SW48-SLC26A9 cell line coculture for 48 h, the cell medium containing serum was replaced for the next experiment. The β-catenin-specific agonist CHIR-99021 at 10 μm/L had the highest agonist effect, and after COLO 201-shSLC26A9 cell line coculture for 48 h, the cell medium containing serum was replaced for the next experiment. Finally, WB was used to detect the expression of related proteins. XAV-939 and CHIR-99021 were obtained from Med-Chem Express (New Jersey, USA).

### Cell nucleus and cytoplasm extraction and cellular immunofluorescence

The nucleus and cytoplasm were extracted using a nuclear and cytoplasmic separation kit. The cells were grown in large petri dishes. Before the experiment, the confluence degree of the cells reached 90%, and the nuclear and cytoplasmic proteins were extracted for protein verification.

To observe the position of SLC26A9 and β-catenin in CRC cells. Briefly, cell slides were first prepared, followed by cellular immunohistochemistry, fluorescent secondary antibodies were added after rewarming the next day, and DAPI staining was used. The slides were imaged with a confocal laser scanning microscope (Zeiss, Jena, Germany).

### Co-Immunoprecipitation (Co-IP)

For Co-IP, cell lysates containing a protease/phosphatase inhibitor mixture (1000 μg) were swirled overnight with SLC26A9 Flag, anti-β-catenin IgG, and rabbit IgG (Beyotime, China) to obtain IP. Then, 30 μl protein A/G Santa Cruz Biotechnology (USA) was added to the lysate and incubated at 4 °C for 4 h. The lysate was washed three times with cracking buffer. Western blotting was used to analyze the IP complex. The primary antibodies used in our study are shown in Supplementary Table 1.

### Xenograft-tumor assay

All animal experiments were conducted with the approval of the Animal Protection and Ethics Committee of the Affiliated Hospital of Zunyi Medical University. The animal study was conducted with reference to the National Institutes of Health’s Guidelines for the Care and Use of Laboratory Animals. BALB/c nude mice (4–5 weeks) were purchased from Beijing SPF (Beijing) Biotechnology Co., Ltd (Beijing, China). A single-cell suspension was prepared, and 2 × 10^6^ cells were inoculated subcutaneously into the two sides of nude mice without the thymus. The tumor formation rate and growth of animals in each group were dynamically observed. After 25 days of inoculation, animals in each group were killed after intravenous injection of barbiturate. The tumor volume was calculated as follows: tumor volume (mm^3^) = 1/2 (length ×width^2^)/2.

### Peptide microarray chip

Peptide microarray chips were synthesized by ChinaPeptides (Shanghai, China). Peptide chip synthesis based on the SLC26A9 protein sequence. AutoSpot peptide synthesizer for synthesizing peptide arrays. For immunological hybridization experiments, the peptide chip was blocked by incubation with reactive protein (recombinant human β-catenin ab 63175, 3 µg/ml) and incubated with primary antibody (anti-β-catenin antibody ab 16051, 10 µg/ml). The peptide array chip was incubated with HRP-labeled antibody (Sheep anti-rabbit HRP, Biyuntian, 1:8000 dilution). ECL (Pierce ECL Western Blotting Substrate, 32109) luminescent reagent was added, shielded from light for 2 min, and imaged with an FX 7 digital imaging analyzer.

### Statistical analysis

The statistical significance of the studies was analyzed by two-tailed Student’s *t* test or one-way ANOVA using SPSS 18.0 software (SPSS Inc., Chicago, IL, USA). Survival curves were plotted using the Kaplan–Meier method and compared using log-rank tests. All analyses were performed at least in triplicate. The results are reported as the means ± SDs. Significance was set at *p < 0.05, **p < 0.01, ***p < 0.001, or ****p < 0.0001; ns not significantly different.

### Supplementary information

Supplementary materials

## Data Availability

All data are available in the main text or the Supplementary Materials.
