# Supplementary material for: SLC26A9 promotes colorectal tumorigenesis by modulating Wnt/β-catenin signaling
Source: Cell Death Discov. 2024 Mar 9;10:123. doi: 10.1038/s41420-024-01888-6 (PMC10925040; doi:10.1038/s41420-024-01888-6)

**
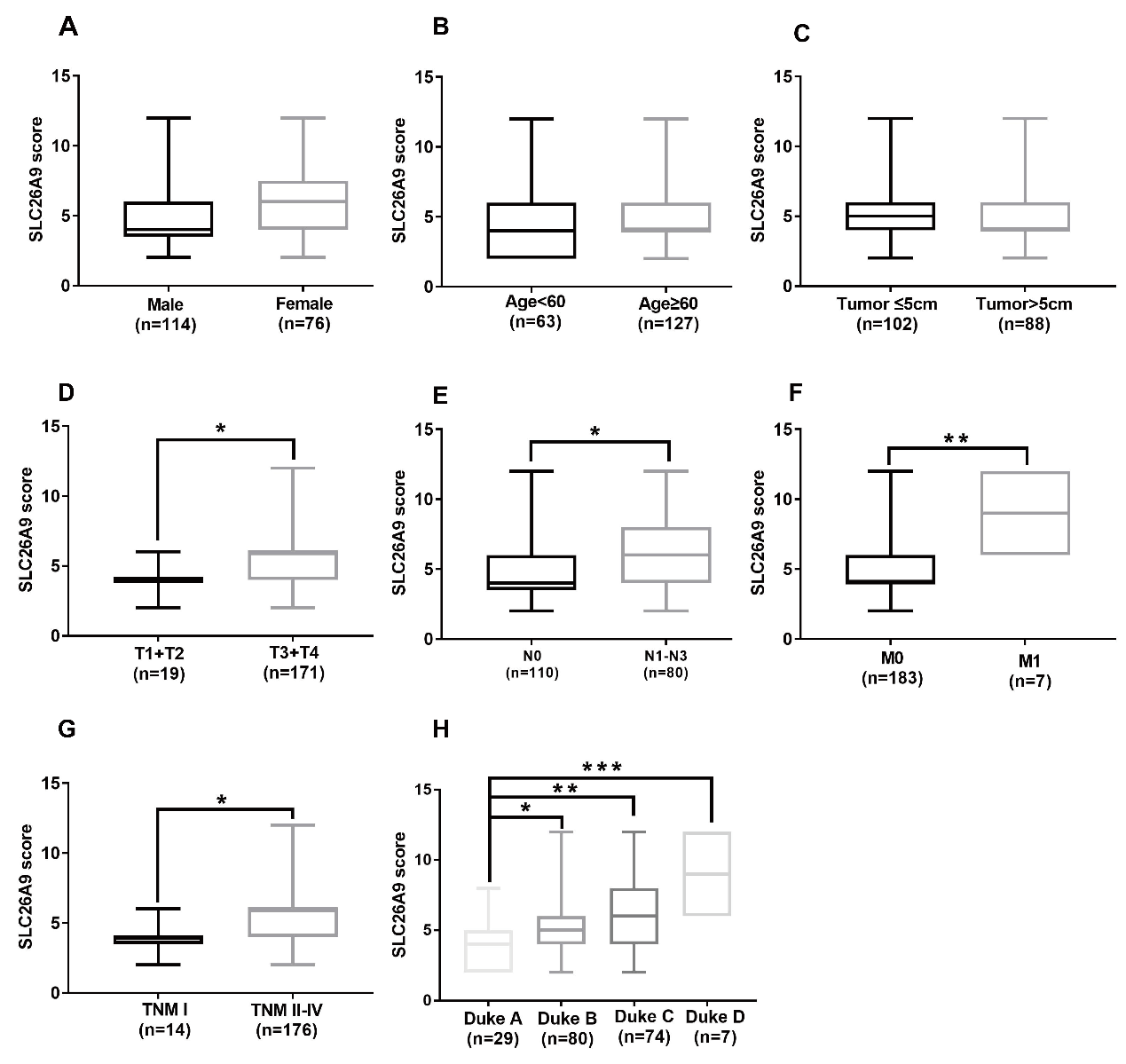
**

**Supplementary Figure 1. Upregulation of SLC26A9 is associated with the development and progression of human CRC and poor prognosis.** SLC26A9 expression in CR tissues under different sex (A), age (B), tumor size (C), stage (D), lymph node (E), metastasis (F), TNM stage (G), and Duke stage (H). **p* < 0.05, ***p* < 0.01, and ****p* < 0.001, compared with relevant controls.


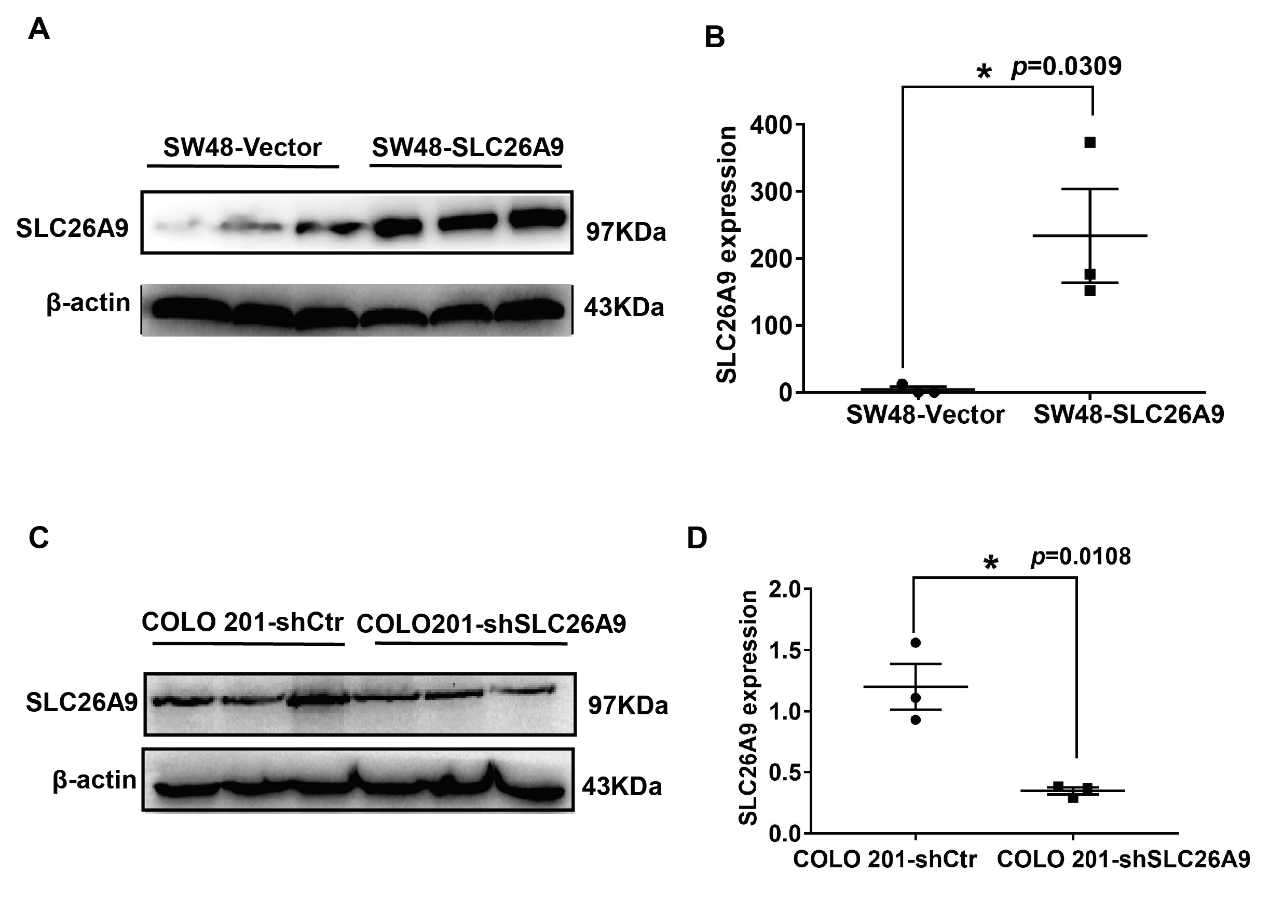


**Supplementary Figure 2. Expression of SLC26A9 in CRC cells.** (A-B) SW48 cells were transfected with lentivirus carrying the SLC26A9 gene fragment or empty vector. A stable strain was selected by puromycin and confirmed by western blot and RT‒qPCR analyses to validate the activation efficiency. (C-D) A stable SLC26A9-silenced strain was constructed in COLO 201 cells. A stable strain was selected by puromycin and confirmed by western blot and RT‒qPCR analyses to validate the repressive efficiency. **p* < 0.05, compared with relevant controls.

**
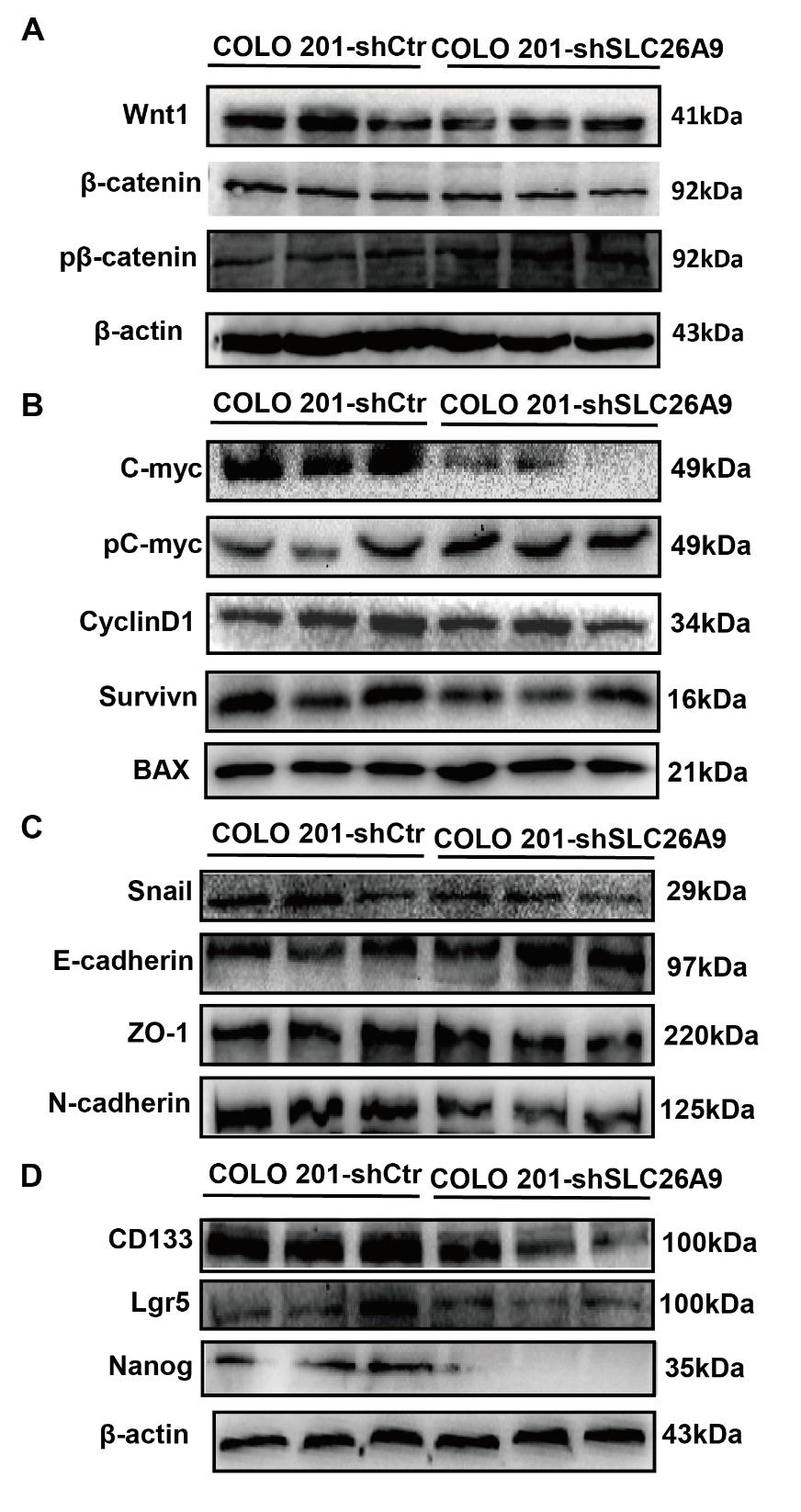
**

**Supplementary Figure 3. The effects of SLC26A9 on the Wnt/β-catenin signaling pathway, proliferation, apoptosis, EMT and CSC phenotypes in COLO201 cells.** (A-D) Multiple markers of the Wnt/β-catenin signaling pathway (A), cell proliferation and apoptosis (B), EMT (C) and CSC phenotypes (D) were detected in COLO201 cells with and without SLC26A9 expression by western blot assay.

**
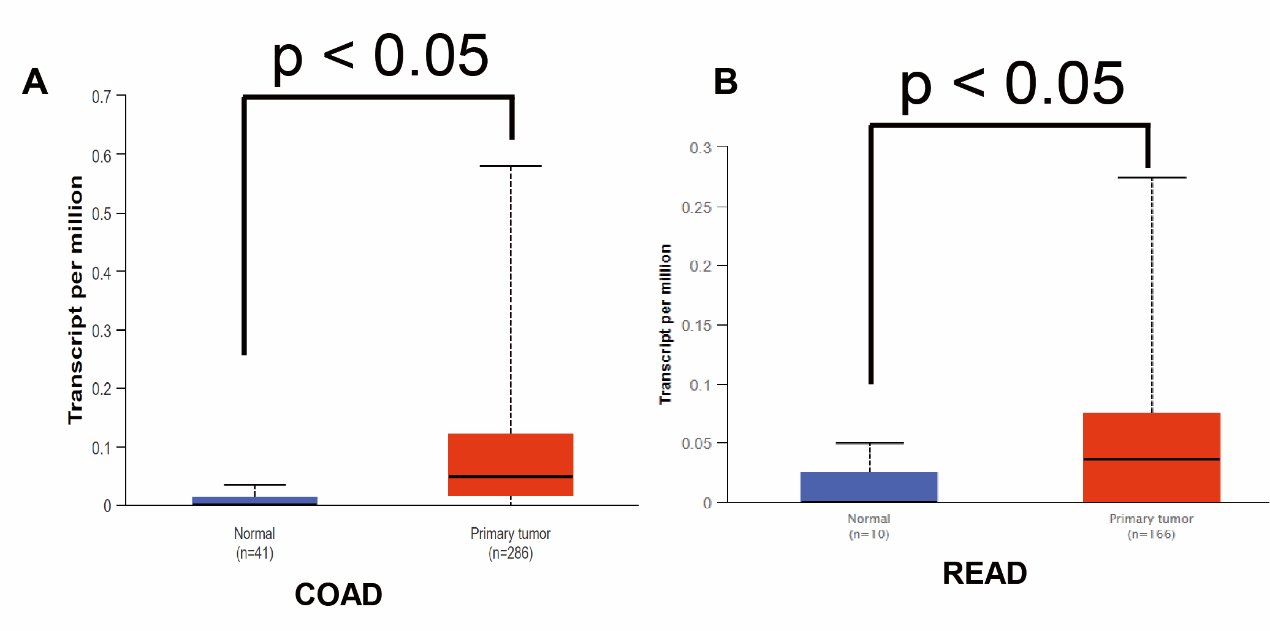
**

**Supplementary Figure 4. SLC26A9 was upregulated in CRC tissues based on the TCGA database.** A. The differential expression of SLC26A9 in colon tissues (n = 286) and normal mucosal epithelial tissues (n = 41) was analyzed based on TCGA; **p* < 0.05, compared to the normal mucosal epithelial tissue group. B. The differential expression of SLC26A9 in rectal tissues (n = 166) and normal mucosal epithelial tissues (n = 10) was analyzed based on TCGA. **p* < 0.05, compared to the normal mucosal epithelial tissue group. COAD: Colon adenocarcinoma; READ: Rectum adenocarcinoma.

**Supplementary Table**

**Supplementary Table 1.** List of antibodies and staining conditions for tissue IHC

| Antibody/Probe | Catalog # | Vendor | Antigen dilution |
| --- | --- | --- | --- |
| **Primary Antibodies to**: | | | |
| SLC26A9 | HPA-051485 | Sigma-Aldrich | 1:50***** |
| SLC26A9 | NBP1-59514 | Novus Biologicals | 1:2000 |
| Wnt1 | ab15251 | Abcam | 1:1000 |
| β-catenin | ab32572 | Abcam | 1:1000,1:200* |
| β-catenin | ab231305 | Abcam | 1:500 |
| p-β-catenin | ab27798 | Abcam | 1:500 |
| N-cadherin | ab18203 | Abcam | 1:1000 |
| E-cadherin | ab40772 | Abcam | 1:500 |
| ZO-1 | 61-7300 | Thermofisher Scietific | 1:500 |
| Fibronectin | ab2413 | Abcam | 1:500 |
| Snail | ab180714 | Abcam | 1:1000 |
| CCND1 | 55506 | Cell Signaling Technology | 1:1000 |
| AIF | ab32516 | Abcam | 1:1000 |
| Caspase 9 | ab202068 | Abcam | 1:2000 |
| c-Myc | bs-0842R | Bioss Antibodies | 1:2000 |
| Cytochrome C | ab133504 | Abcam | 1:5000 |
| CD44 | ab157107 | Abcam | 1:1000 |
| Cleaved Caspase-3 | #9661 | Cell Signaling Technology | 1:2000 |
| CD133 | ab19898 | Abcam | 1:1000 |
| Flag-Tag | T9953 | Affinity Research | 1:25000 |
| β-actin | #HC201 | TransGen Biotech | 1:5000 |
| GAPDH | #HC301-01 | TransGen Biotech | 1:5000 |
| Ki-67 | ab8191 | Abcam | 1:50* |
| Lrg5 | abs120810 | Absin | 1:400 |
| H3 | ab1791 | Abcam | 1:5000 |

List of antibodies was used in this study, * For IHC, others for Western Blot

**Supplementary Table 2.** List of primers of multiple genes was used in this study

| **RT-qPCR** | | |
| --- | --- | --- |
| **Gene (Human)** | **Forward sequence** | **Reverse sequence** |
| SLC26A9 | CCCAGGACACAACTTCCAA | AGGCACTCTGTAGGCAGCAT |
| β-actin | TGGCACCCAGCACAATGAA | CTAAGTCATAGTCCGCCTAGAAGCA |

COLO 201 by STR profiling


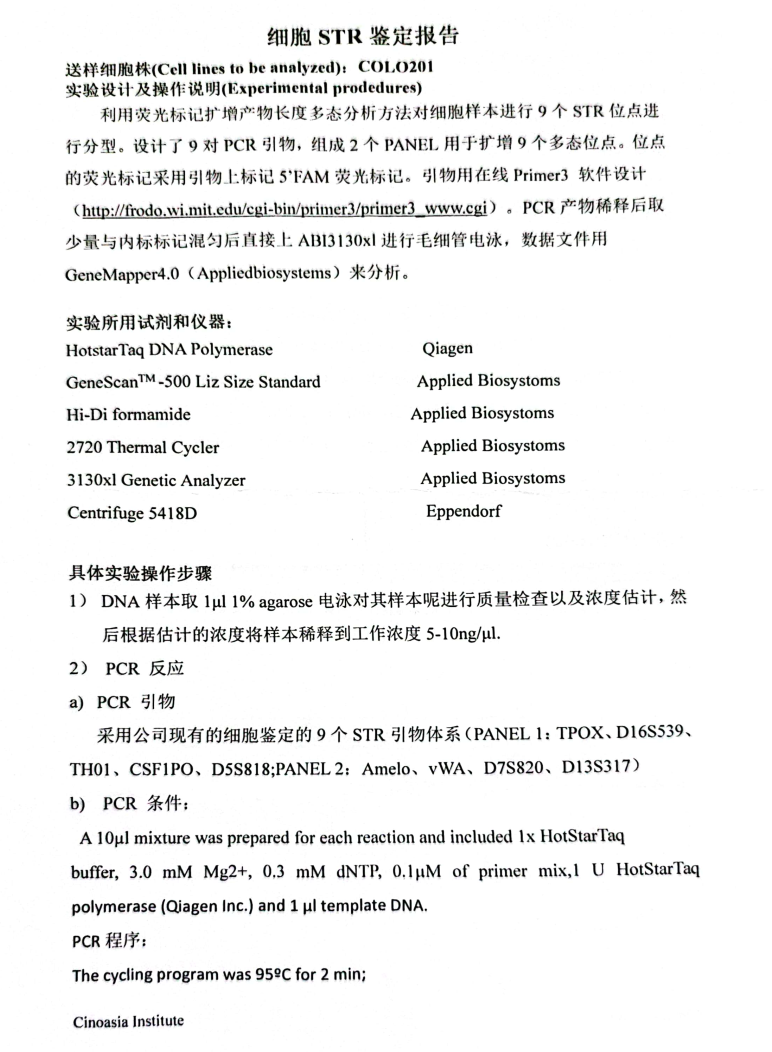


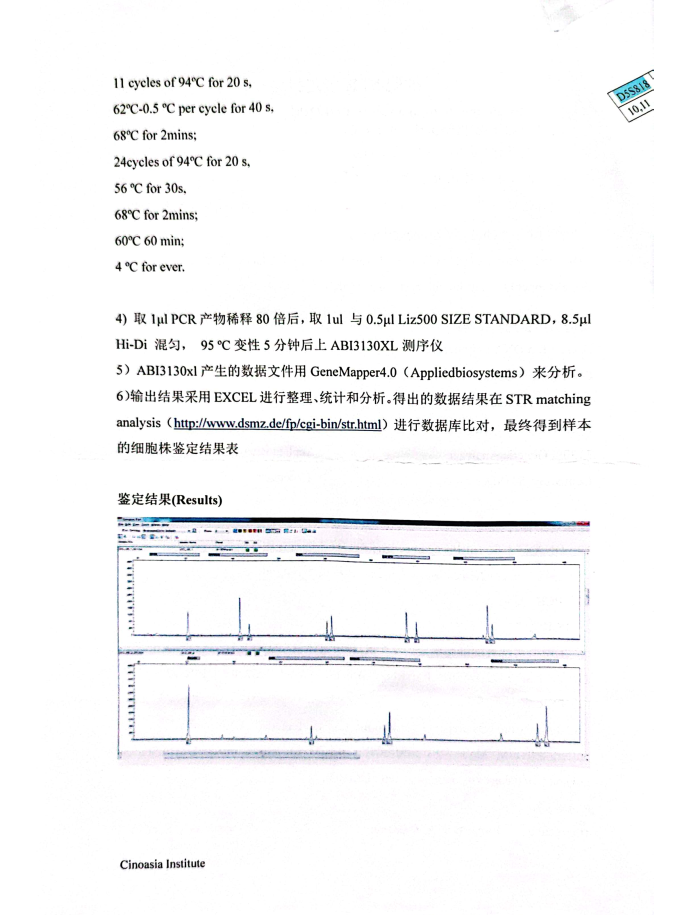


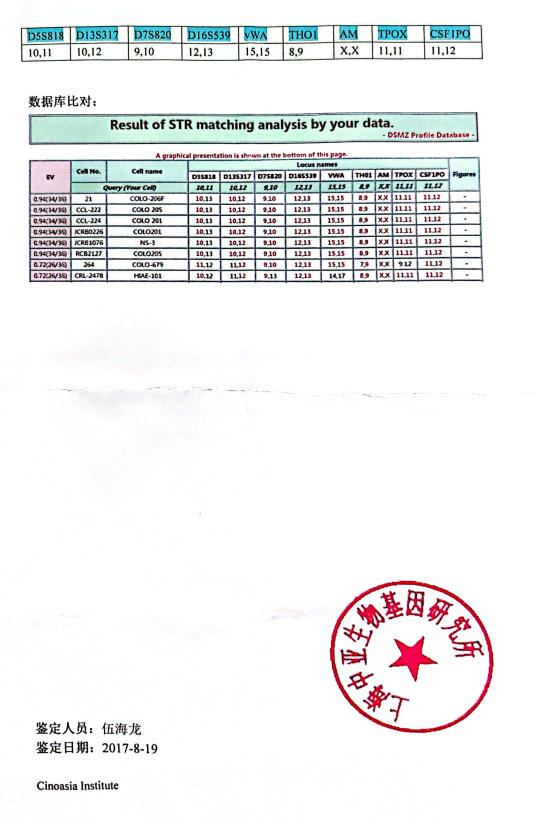


SW48 by STR profiling


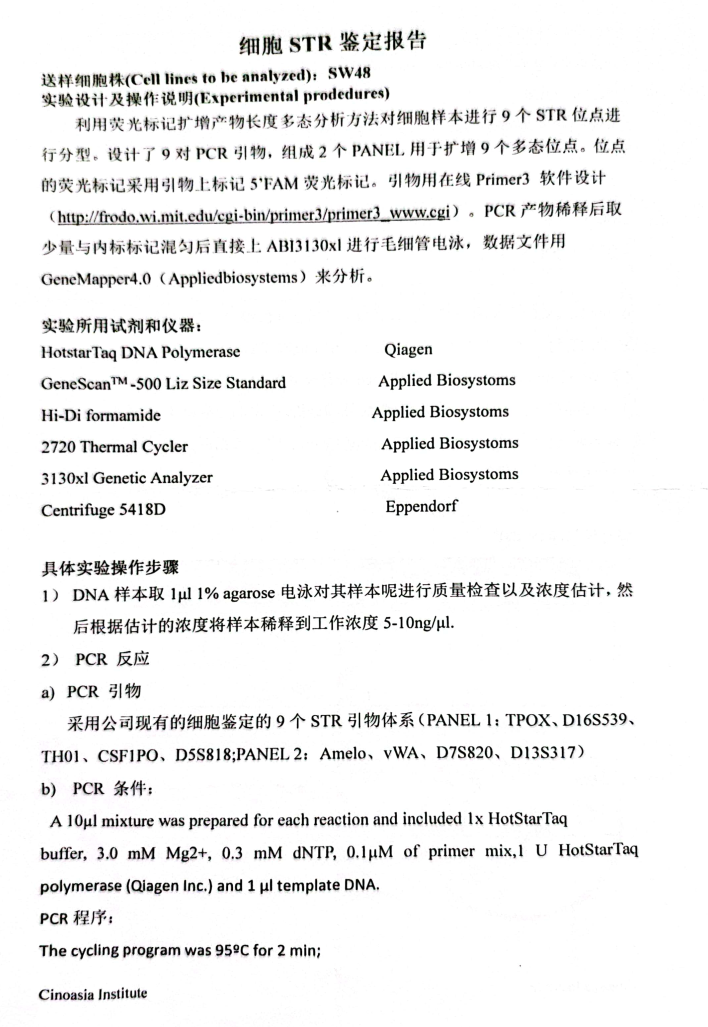


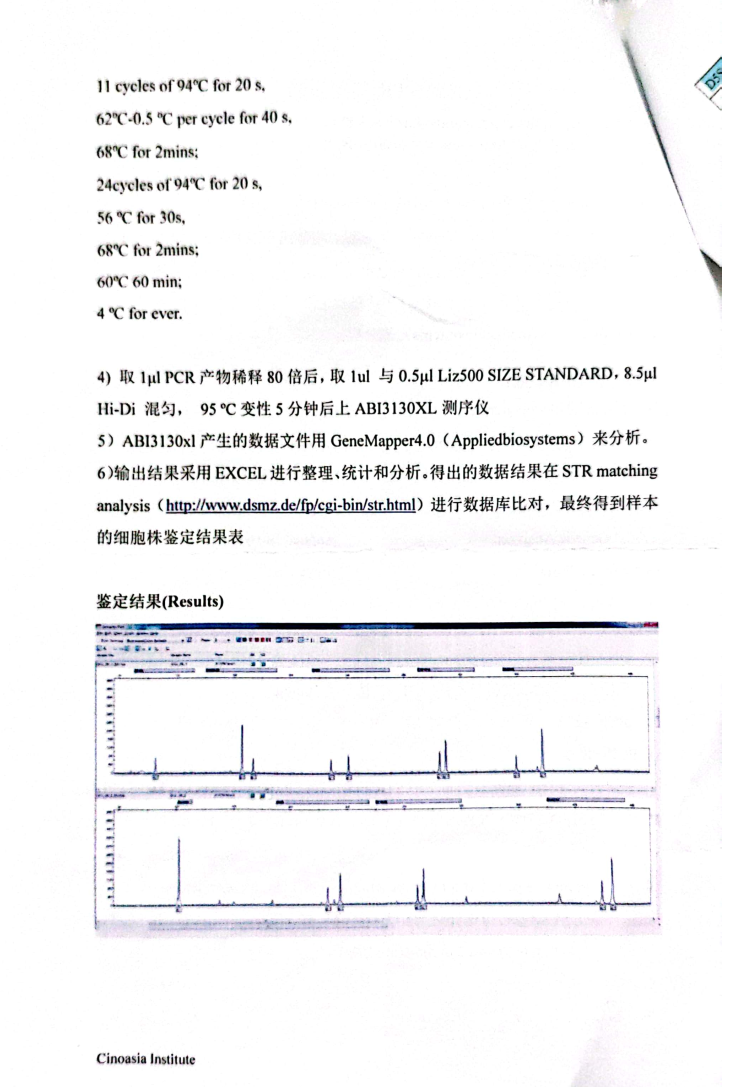


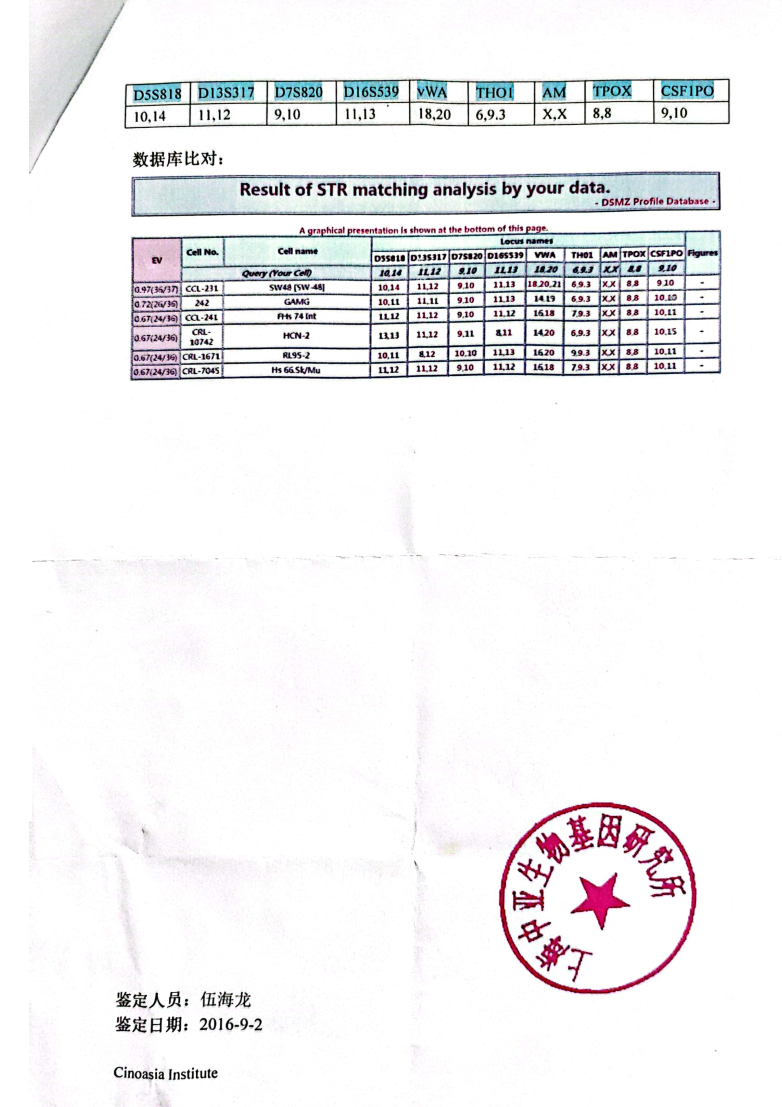

Supplement: Supplementary file 1 — Supplementary materials [file 41420_2024_1888_MOESM1_ESM.docx]
